# Acupuncture for amnestic mild cognitive impairment: Study protocol for a multicenter, single-blinded, long-term, randomized controlled trial

**DOI:** 10.1371/journal.pone.0346717

**Published:** 2026-04-20

**Authors:** Xinyue Zhang, Ziwen Wang, Mingsheng Sun, Qiongnan Bao, Ziwen Chen, Yiwei Liu, Ziqi Wang, Fang Ye, Zuoqin Yang, Xiaoli Du, Hong Zhang, Xin Mou, Xia He, Dehua Li, Kexin Wu, Jin Yao, Wanqi Zhong, Ping Xu, Shiqi Yang, Ling Zhao, Zihan Yin, Fanrong Liang

**Affiliations:** 1 School of Acu-Mox and Tuina, Chengdu University of Traditional Chinese Medicine, Chengdu, China; 2 Sichuan Provincial Acupuncture Clinical Medicine Research Center, Chengdu, China; 3 The West China Hospital, Chengdu, China; 4 The Fourth People’s Hospital of Chengdu, Chengdu, China; 5 The Sichuan Province People’s Hospital, Chengdu, China; 6 Traditional Chinese Medicine Hospital of Pidu district, Chengdu, China; 7 The Nanchong Second People’s Hospital, Nanchong, China; 8 Traditional Chinese Medicine Hospital of Meishan, Meishan, China; 9 Chengdu First People’s Hospital, Chengdu, China; 10 The Rehabilitation Hospital of Sichuan Province, Chengdu, China; 11 Hospital of Chengdu University of Traditional Chinese Medicine, Chengdu, China; Beijing University of Chinese Medicine, CHINA

## Abstract

**Background:**

Amnestic mild cognitive impairment (aMCI), a common neurodegenerative disease affecting older adults, has garnered significant research interest over the past few years. While previous studies have suggested that acupuncture holds promise as a clinical intervention to improve cognitive function in patients with aMCI, the long-term effect of acupuncture treatment for aMCI remains unclear.

**Methods:**

This is a multicenter, single-blinded, randomized controlled trial (RCT) with a long-term follow-up.166 patients diagnosed with aMCI will be randomly divided into acupuncture group (AG) and sham acupuncture group (SA). The intervention will last for 12 weeks (2 sessions per week), follow-up for 48 weeks, and the study will last a total of 60 weeks. The primary outcomes are the changes in the Alzheimer’s Disease Assessment Scale-Cognitive (ADAS-Cog) score from baseline to week 12 and from baseline to week 60. Generalized estimating equations (GEE) will be used to assess the impact of the acupuncture intervention on outcome variables over time at baseline and weeks 12, 24, 48, and 60.

**Discussion:**

This protocol outlines a detailed procedure for a multicenter RCT designed to further evaluate the long-term effect of acupuncture in managing aMCI. We anticipate that the findings of this research will provide valuable insights and evidence-based recommendations for the clinical management of this patient population.

**Trial registration:**

The trial was registered at Chinese Clinical Trial Registry on 28 May 2024 (Number: ChiCTR2400084932).

## Introduction

Amnestic mild cognitive impairment (aMCI) is a common neurodegenerative disease affecting older adults and is also the most prevalent subtype of mild cognitive impairment (MCI), accounting for approximately two-thirds of all cases. The hallmark clinical features include memory decline and impaired cognitive function, while individuals generally maintain their ability to perform daily activities. A meta-analysis study from 2022 showed that the prevalence rate of MCI in people over 50 years old was 15.56% globally, of which the prevalence rate of aMCI was 10.03% [1]. Moreover, the prevalence rate increased with age. A report released by the World Health Organization in 2019 showed that Alzheimer’s disease (AD) and other types of dementia have become the seventh leading cause of death [1]. However, the pathogenesis of AD is complex and the therapeutic effect is general [2]. aMCI represents a transitional state between AD and normal aging [3–5], and its risk of progression to AD is about 40% [6]. Given that aMCI is the early stage of AD and the large number of patients, early intervention of aMCI is particularly necessary [7].

However, the pathogenesis of aMCI remains elusive. While it shares some pathophysiological mechanisms with AD, there are numerous hypotheses regarding its origins [8–13]. Currently, there are no approved drugs that effectively manage aMCI and improve cognitive function [14]. Studies have demonstrated that existing medications are not only ineffective in controlling aMCI [15,16] but also frequently cause adverse reactions like nausea, diarrhea, and vomiting [17]. Concerningly, projections indicate that by 2050, the annual cost of treating cognitive impairment in China alone will reach 1.89 trillion yuan, with the global cost reaching an astounding USD 9.12 trillion [18]. This underscores the severe economic burden that aMCI places on individuals, families, and society. Given that aMCI is a potential precursor to AD, the development of safe, effective, and affordable treatments is of paramount importance.

Acupuncture, a commonly used and well-tolerated complementary and alternative therapy, has experienced a steady rise in popularity for treating aMCI [19]. Evidence-based research suggests that it is an effective clinical approach for improving cognitive function in aMCI patients [20–22]. We have completed a preliminary clinical trial on acupuncture intervention in aMCI patients [23], demonstrating lasting but unclearly sustained positive effects. Acupuncture is a complex intervention [24], and the placebo effect of acupuncture is controversial [25–27]. We will introduce the sham acupuncture group in the control arm. This ensures comparable credibility to true acupuncture while minimizing actual stimulation [25]. Additionally, aMCI is a chronic disease, and its impact on health and life is long-standing. We believe an extended follow-up is necessary to elucidate the long-term efficacy. To further solidify our findings and establish generalizability, we propose conducting a large-scale, sham-acupuncture controlled, long-term follow-up clinical trial.

### Objective

(1) To evaluate the efficacy and safety of acupuncture compared to sham acupuncture in the treatment of patients with aMCI; (2) To determine the long-term effect of acupuncture in the treatment of aMCI patients.

## Methods

This study employs a randomized controlled trial design. The protocol adheres to the Standard Protocol Items: Recommendations for Interventional Trials (SPIRIT) [28] (S1 File). This study is registered in the Chinese Clinical Trials Register (ChiCTR2400084932) and will be conducted in strict compliance with the Declaration of Helsinki. It has received approval from the Medical Ethics Committee of Hospital of Chengdu University of Traditional Chinese Medicine (ethical approval number: 2023KL-144). The study recruitment began on June 30, 2024 and is scheduled to conclude in December 2026. Data collection will end in March 2027, and data analysis will be completed in June 2027.

### Study design

This prospective, multicenter randomized controlled trial (RCT) will employ a 1:1 allocation ratio with concealed allocation to assign 166 patients with aMCI evenly into either the acupuncture group (AG) or sham acupuncture group (SA) randomly. The intervention will last for 12 weeks (2 sessions per week), followed by a 48-week follow-up period, for a total observation period of 60 weeks.

**Fig 1:**
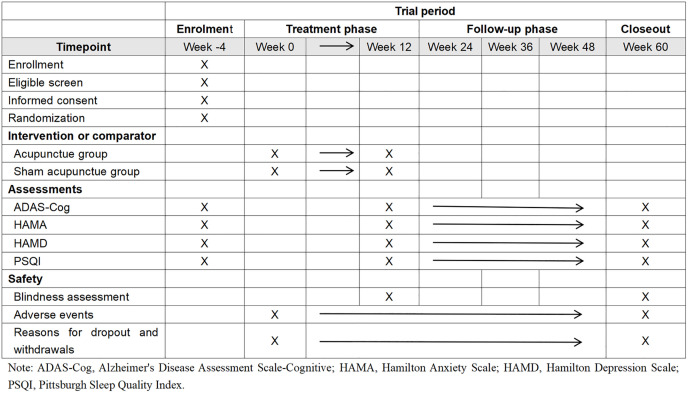
A flow chart of the trial.

**Fig 2:**
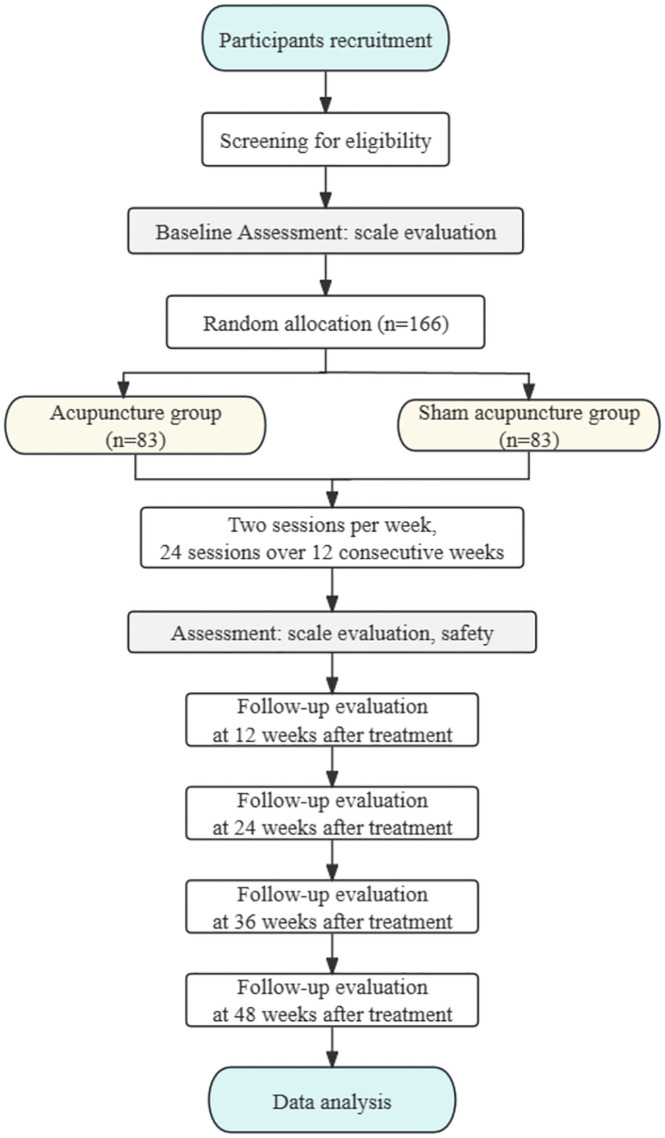
CONSORT 2010 flow diagram.

**Fig 3:**
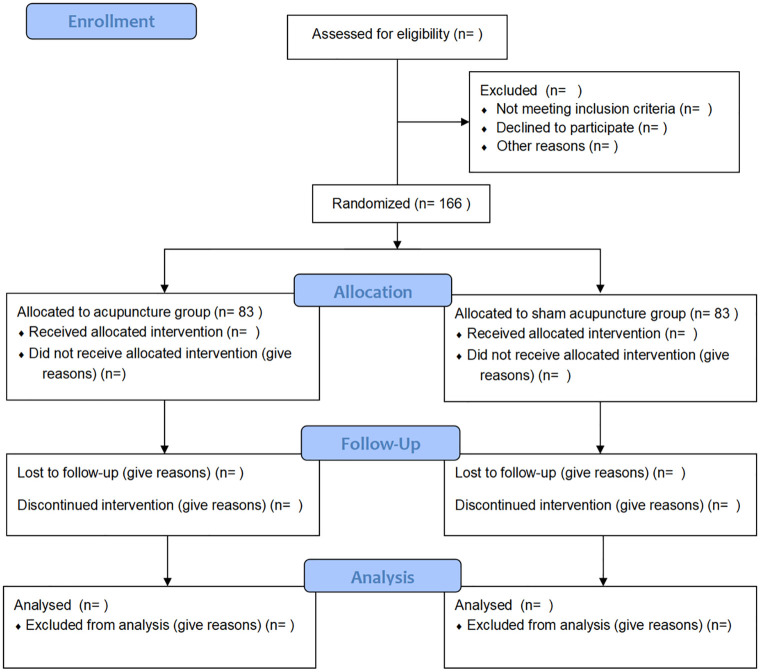
SPIRIT schedule.

The trial will be conducted across the outpatient clinics of nine hospitals in China (the West China Hospital, the Fourth People’s Hospital of Chengdu, the Sichuan Province People’s Hospital, Traditional Chinese Medicine Hospital of Pidu district, the Nanchong Second People’s Hospital, Traditional Chinese Medicine Hospital of Meishan, Chengdu First People’s Hospital, the Rehabilitation Hospital of Sichuan Province and Hospital of Chengdu University of Traditional Chinese Medicine). Recruitment will involve social media advertisements and postings at community centers. Research assistants will obtain written informed consent from willing participants before baseline assessment, and data collectors will remain blinded to group assignments. Figs 1–3 illustrate the research procedure and results evaluation schedule.

### Eligibility criteria

#### Inclusion criteria.

Patients who meet 8 following criteria will be included: (1) a diagnosis of aMCI according to the Jak/Bondi 2014 criteria [29]; (2) age between 50 and 80 years, regardless of sex; (3) a disease course≥ 3 months; (4) a Clinical Dementia Rating (CDR) score of 0.5; (5) a Hachinski Ischemic Score (HIS)≤4; (6) ≥8 years of education (including vocational) with the ability to understand and complete scales; (7) willingness to participate and provide signed informed consent; (8) no contraindications for magnetic resonance imaging (MRI) scanning.

#### Exclusion criteria.

Patients will be excluded if they meet any of the following criteria: (1) receiving treatment that interferes with cognitive function (e.g., the treatment of acute psychotic illness, such as donepezil, memantine, oxiracetam, sodium oligomannate capsules, and rivastigmine); (2) a history of neurological conditions affecting cognitive function confirmed by examination, except in patients with suspected early AD (including vascular dementia, Parkinson’s disease, traumatic brain injury, brain tumor or other diseases which might lead to neurological injury and abnormal brain structure). Presence of systemic diseases that could cause cognitive decline (such as hepatic encephalopathy, Hashimoto’s encephalopathy, metabolic encephalopathy, renal encephalopathy, and anemia); (3) the brain MRI showing infection or other local lesions, infarction in vital memory brain areas, multiple embolic infarctions, or severe white matter lesions (Fazekas score≥3); (4) a history of psychiatric illness (e.g., bipolar disorder, schizophrenia), severe anxiety (Hamilton anxiety [HAMA] scale score≥29), major depression (Hamilton Depression [HAMD] scale score≥24), or tumor; (5) severe skin infection, hemorrhagic disease, or bleeding tendency; (6) severe drug dependence, drug, smoking, or alcohol abuse; (7) pregnant, potentially pregnant, or lactating females; and (8) receipt of any acupuncture treatment or participation in other clinical trials within 6 months before enrollment.

#### Withdrawal criteria.

Patients will be informed that they can withdraw from the study at any time. However, the researchers will ask for and record the reason for withdrawal. Patients will be withdrawn when the following conditions occur: (1) patients who do not meet the inclusion criteria and were mistakenly enrolled; (2) patients who utilize prohibited treatments or independently modify their treatment plan as outlined in the protocol; (3) patients who experience significant life events or circumstances that make continued participation unsuitable for their well-being.

### Randomization

After baseline evaluation, eligible patients will be randomly assigned to either the AG or SA group in a 1:1 ratio using computer-generated random numbers. Before randomization, all participants will be informed of their potential allocation to either group. The allocation sequence will be concealed using sealed, opaque, sequentially numbered envelopes, which will only be opened upon enrollment of eligible participants. Independent statisticians, uninvolved in participant recruitment, evaluation, and intervention, will be responsible for the sequence generation and allocation process throughout the study. The detailed standard operating procedure for envelope management is provided in S4 File.

### Blinding

This study will employ the single-blinded method. Patients will be treated separately to ensure blind evaluation. A third-party assessors who do not know group assignments will assess outcomes. Due to the unique nature of acupuncture procedures (including sensations and depth), it will not be possible to blind the acupuncturists. However, all other research personnel, including research fellows, outcome assessors, and statisticians, will be blinded to randomization results,study procedure and any treatment interventions for the entire study duration. To maintain consistency, each patient will be assessed by the same evaluator. When participants complete all 24 acupuncture sessions, they will be asked whether they received real or sham acupuncture to assess blinding effects. Efficacy evaluators and statisticians will be separated and blinded. Additionally, at the 12-week and 60-week time points, a simplified blinding assessment questionnaire will be administered to all outcome assessors. The questionnaire will inquire about their guess regarding the group assignment (AG, SA, or unclear) of the evaluated participant. The responses will be analyzed using the James’ blinding index to objectively evaluate the maintenance of blinding.

### Sample size

The Alzheimer’s Disease Assessment Scale-Cognitive (ADAS-Cog) score will serve as the primary index to evaluate cognitive function improvement in patients with MCI. The sample size calculation for this trial was based on our previous study [30] and pilot study, we anticipate an average effect of 3.5 in the acupuncture group and 1.5 in the control group, with a standard deviation of 3.5 between the groups. Using PASS software, we calculated a required sample size of 132 subjects (α = 0.05, 1 − β = 0.90, two-sided). To account for a potential 20% loss to follow-up, we plan to enroll 83 patients in each group, for a total of 166 aMCI patients in the study.

### Interventions

Both groups will receive the same number of acupuncture sessions, with identical treatment frequency and duration. To ensure standardization, all interventions will be performed by licensed Traditional Chinese Medicine acupuncturists with more than 6 years of experience, and these practitioners will administer both real and sham acupuncture.

#### Acupuncture group.

Acupuncture treatment will adhere to the Standards for Reporting Interventions in Controlled Trials (STRICTA) [31] (**Table 1**). The following acupoints will be used: bilateral Taixi (KI 3), bilateral Dazhong(KI 4), bilateral Sanyinjiao (SP 6), Shenting (GV 24), and Baihui (GV 20) (**Table 2**). Licensed acupuncturists will follow TCM standards for acupoint location and manipulation. After skin disinfection, acupuncturists will insert single-use sterile needles (Hwato, Suzhou, China; 0.25 × 25 mm) into the acupoints. A uniform reinforcing-reducing method will be employed, involving twisting, thrusting, and rotation to elicit the *deqi* sensation within the patient’s tolerance. Needles will be manually manipulated every 10 minutes to maintain the *deqi* sensation, with a twisting angle of 90–180 degrees, a frequency of 60–90 times/minute, and a lifting and inserting amplitude of 3–5 mm. Treatment sessions will last 30 minutes and continue for 12 weeks, 2 sessions per week, each interval of 2–3 days.

**Table 1:** Acupuncture treatment details based on the STRICTA 2010 checklist.

Item	Item number	Detail
1. Acupuncture rationale	(1a) Style of acupuncture	Traditional Chinese Medicine
	(1b) Reasoning for treatment provided, based on historical context, literature sources, and/or consensus methods, with references where appropriate	The treatment is carried out according to traditional acupuncture theory, previous studies, and the experts’ consensus.
	(1c) Extent to which treatment was varied	Standardised acupuncture treatment.
2. Details of needling	(2a) Number of needle insertions per subject per session	8
	(2b) Names of points used	Bilateral Taixi (KI 3), bilateral Dazhong(KI 4), bilateral Sanyinjiao (SP 6), Shenting (GV 24), and Baihui (GV 20).
	(2c) Depth of insertion, based on a specified unit of measurement, or on a particular tissue level	From 0.3–1 cun.
	(2d) Response sought	*Deqi* (numbness, soreness, heaviness, distention, etc.)
	(2e) Needle stimulation	Manual acupuncture
	(2f) Needle retention time	30 min
	(2g) Needle type	Sterile, disposable acupuncture needles (length, 25 mm; diameter, 0.25 mm; Hwato, China).
3. Treatment regimen	(3a) Number of treatment sessions	24
	(3b) Frequency and duration of treatment sessions	Twice per week (once per 2–3 days interval), for 12 continuous weeks.
4. Other components of treatment	(4a) Details of other interventions administered to the acupuncture group	/
	(4b) Setting and context of treatment, including instructions to practitioners, and information and explanations to patients	The trial will be implemented at departments in the outpatient clinics of nine hospitals (the West China Hospital, the Fourth People’s Hospital of Chengdu, the Sichuan Province People’s Hospital, Traditional Chinese Medicine Hospital of Pidu district, the Nanchong Second People’s Hospital, Meishan Hospital of traditional Chinese Medicine, Chengdu First People’s Hospital, the Rehabilitation Hospital of Sichuan Province and Hospital of Chengdu University of Traditional Chinese Medicine). All information and explanations will be provided to participants.
5. Practitioner background	(5) Description of participating acupuncturists	Trained, licensed acupuncturists with at least 6 years in acupuncture clinical practice.
6. Control or comparator interventions	(6a) Rationale for the control or comparator in the context of the research question, with sources that justify this choice	The treatment is carried out according to previous studies, and the experts’ consensus.
	(6b) Precise description of the control or comparator. If sham acupuncture or any other type of acupuncture-like control is used, provide details as for Items 1–3 above.	Sham acupuncture will be performed at 8 non-acupoints.
	(6b-1) Style of acupuncture	The sham acupuncture is invasive (penetrating the skin).
	(6b-2) Reasoning for treatment provided, based on historical context, literature sources, and/or consensus methods, with references where appropriate	The protocol for choosing the non-acupoints was developed in our previous clinical trial.
	(6b-3) Extent to which treatment was varied	Standardised sham acupuncture treatment.

**Table 2:** Details of the intervention in the acupuncture group.

Acupoint	Location	Manipulation
Taixi (KI 3)	Posterior to the medial malleolus, in the depression between tip of the medial malleolus and tendo calcaneus.	Subcutaneous insertion to a depth of 0.5–1 cun with manipulation for the *deqi.*
Dazhong (KI 4)	Under the medial malleolus of the foot, the medial anterior depression of the achilles tendon attachment.	Subcutaneous insertion to a depth of 0.3–0.5 cun with manipulation for the *deqi.*
Sanyinjiao (SP 6)	On the inside of the calf, 3 cun above the tip of the medial malleolus, posterior border of the medial border of the tibia.	Subcutaneous insertion to a depth of 0.5–1 cun with manipulation for the *deqi.*
Shenting (GV 24)	0.5 cun directly above the midpoint of the anterior hairline.	Subcutaneous insertion to a depth of 0.3–0.5 cun with manipulation for the *deqi*.
Baihui (GV 20)	5 cun directly above the midpoint of the anterior hairline, at the midpoint of the line connecting the apexes of the two auricles.	Subcutaneous insertion to a depth of 0.5–1 cun with manipulation for the *deqi.*

Note: 1 cun (≈25 mm) is defined as the width of the interphalangeal joint of the patient’s thumb.

#### Sham acupuncture group.

**Fig 4:**
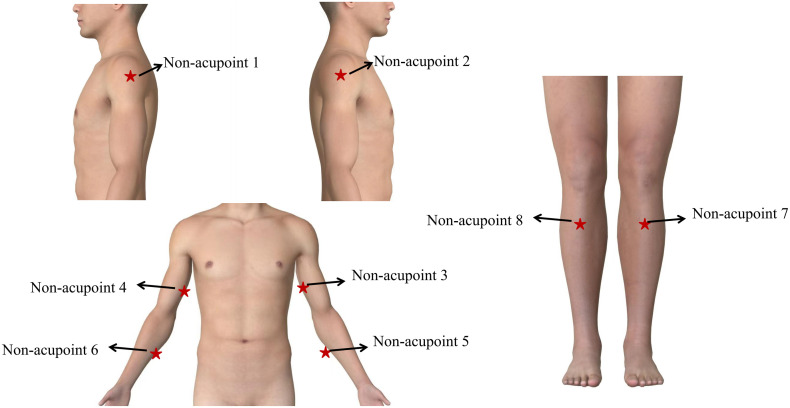
The non-acupoints of sham acupuncture group.

The sham acupuncture intervention will adhere to the Acupuncture Controls guideline for Reporting human Trials and Experiments (ACURATE) [32,33] (**Table 3**). It will involve shallow acupuncture 0.3–0.5 cun, inserted perpendicularly) at eight non-acupoints. The non-acupoint selection protocol was developed in our previous clinical trial [34] (**Table 4 and Fig 4**). Skin disinfection will precede needle insertion. However, there will be no stimulation, manipulation, or attempts to elicit the *deqi* sensation. Licensed acupuncturists will use single-use sterile needles (Hwato, Suzhou, China; 0.25 × 13 mm) for all procedures. Treatment sessions will last 30 minutes and continue for 12 weeks, 2 sessions per week, each interval of 2–3 days.

**Table 3:** Sham acupuncture intervention details based on the ACURATE checklist.

Category	Item	Description	Detail
1. Type of sham acupuncture	1a	Report the type of sham acupuncture.	Sham acupuncture will be performed at four non-acupoints (Shallow acupuncture, 0.3–0.5 cun.)
	1b	Report whether the sham acupuncture is penetrating or non-penetrating.	Penetrating
	1c	Rationale for using the chosen sham acupuncture.	Based on our previous clinical trial [34].
2. Details of sham acupuncture manipulation	2a	Report the number of sham acupuncture applied per subject per session.	8
	2b	Report the depth of sham acupuncture insertion (if there was no penetration, state this within the paper).	0.3-0.5 cun.
	2c	Report whether any response was observed during sham acupuncture manipulation (e.g., de qi or muscle twitch response).	Without attempting to yield the *deqi* sensation.
	2d	Report if there was any stimulation using sham acupuncture.	Without stimulation or manipulation.
	2e	Report if there was sham acupuncture retention.	30 min sessions.
	2f	Report details of other interventions administered in addition to sham acupuncture during one session.	Patients will be allowed to perform basic treatments such as blood pressure control, blood sugar control, and other supportive treatments.
3. Location of sham acupuncture	3a	Report the location of sham acupuncture (e.g., acupoint/non-acupoint or the exact location of the sites).	8 non-acupoints (Table 4 and Fig 4).
	3b	Explicitly state in the paper if the points are unilateral or bilateral.	8 non-acupoints on the left and right sides.
	3c	Describe the reason for the chosen location of sham acupuncture.	Based on our previous clinical trial.
4. Treatment regimen	4a	Report the number of treatment sessions.	30 min sessions over 12 weeks.
	4b	Report whether the number of sessions were identical between real and sham acupuncture treatments.	Both groups will be administered with the same, number of sessions, frequency and treatment duration.
	4c	Report the frequency and duration of treatment sessions.	30 min sessions over 12 weeks.
	4d	Report the total trial period.	12 weeks.
5. Practitioner	5a	Report whether the same practitioner is administering both real and control treatments (interventions).	All interventions will be performed by doctors of Traditional Chinese Medicine with more than 6 years of experience. They will administer both real and sham acupuncture.
	5b	Report whether there were conversations between practitioner and patient directly linked to the trial design, other than scripted instructions and preset information, prior to and during the treatment.	Throughout the course of treatment, acupuncturists will be asked to avoid discussing treatment options with patients.
6. Protocol and settings	6a	Report the information regarding sham acupuncture provided to participants.	Before randomization all participants will be informed that they will be allocated to the acupuncture group or sham acupuncture group.
	6b	Report whether the information given to patients include the term to openly state that the control is inert (e.g., “fake”, “sham”, “dummy”,”placebo”,...).	The participants will be informed that they will receive one of the two acupuncture treatments.
	6c	Describe how sham device was blinded from patients, and if done, how the blinding was assessed.	Patients will be treated separately to ensure blind evaluation.
	6d	If done, report any modification in the sham acupuncture treatment procedure, and reason for the modification.	Not Applicable.
	6e	Report any difference in the treatment settings between real and sham acupuncture.	The choice of acupoints and the depth of insertion varied.

Note: 1 cun (≈25 mm) is defined as the width of the interphalangeal joint of the patient’s thumb.

**Table 4:** Details of the intervention in the sham acupuncture group.

Non-acupoint	Location	Manipulation
Non-acupoint 1	At the medial arm on the anterior border of the insertion of the deltoid muscle at the junction of deltoid and biceps muscles. (On the right arm)	Punctured perpendicularly 0.3-0.5 cun.
Non-acupoint 2	At the medial arm on the anterior border of the insertion of the deltoid muscle at the junction of deltoid and biceps muscles. (On the left arm)	
Non-acupoint 3	Half way between the tip of the elbow and axillae. (On the right arm)	
Non-acupoint 4	Half way between the tip of the elbow and axillae. (On the left arm)	
Non-acupoint 5	Ulnar side, half way between the epicodylus medialis of the humerus and ulnar side of the wrist. (On the right arm)	
Non-acupoint 6	Ulnar side, half way between the epicodylus medialis of the humerus and ulnar side of the wrist. (On the left arm)	
Non-acupoint 7	Edge of the tibia 1–2 cm lateral to the Zusanli (ST36) horizontally. (On the right leg)	
Non-acupoint 8	Edge of the tibia 1–2 cm lateral to the Zusanli (ST36) horizontally. (On the left leg)	

Note: 1 cun (≈25 mm) is defined as the width of the interphalangeal joint of the patient’s thumb.

#### Basic interventions.

During the clinical trial, patients will be allowed to continue receiving basic treatments, such as those for blood pressure control, blood sugar control, or other supportive medications. The details of these treatments, including the time of administration, dosage, frequency, and any reactions experienced by the patient, will be meticulously recorded.

### Outcomes

#### Primary outcome measures.

Cognitive function will be measured using the ADAS-Cog scale [35]. The primary outcomes will be the changes in the ADAS-Cog score from baseline to week 12 and from baseline to week 60. The ADAS-Cog, comprised of 12 tasks, evaluates various aspects of cognition, including memory, language, praxis (ability to perform actions), orientation, and attention. It is a widely used instrument in dementia research, with lower scores indicating less cognitive impairment.

#### Secondary outcome measures.

The secondary outcome measures include the following: (1) The overall cognitive function will be measured using the ADAS-Cog scale, assessed at the12^th^, 24^th^, 36^th^, 48^th^, and 60^th^ weeks; (2) The memory function will be measured using the word recall and word recognition improvement tasks of ADAS-Cog scales at the12^th^, 24^th^, 36^th^, 48^th^, and 60^th^ weeks; (3) The emotional disorders will be measured using the HAMD and HAMA scales [36,37] at the 12^th^, 24^th^, 36^th^, 48^th^, and 60^th^ weeks. The HAMD scale comprises 17 items that measure somatic and affective symptoms of depression, while the HAMA scale comprises 14 items to measure anxiety. Each item of HAMD and HAMA scales is scored for severity on a scale of 0–4, with a higher score reflecting higher symptom severity; and (4) The sleep quality will be measured using the Pittsburgh Sleep Quality Index (PSQI) [38] at the12^th^, 24^th^, 36^th^, 48^th^, and 60^th^ weeks. The questionnaire covers seven components: quality of sleep, the latency of sleep, duration of sleep, the efficiency of sleep, sleep disturbances, use of sleeping drugs, and daytime dysfunction. The PSQI with higher scores indicates the worse sleep quality.

### Safety measurements

Throughout the intervention period, patients will be closely monitored for adverse events (AEs). This includes routine physical examinations and vital sign monitoring for all participants. The research team will implement all necessary measures to prevent potential accidental injuries associated with the study. Should any such injuries occur, the project will cover the related medical expenses. Comprehensive documentation will be maintained for any AEs, including immediate provision of appropriate medical care to affected individuals and a thorough investigation to determine the cause of the events. As acupuncture can potentially cause AEs like bleeding, hematoma, fainting, severe pain, or local infection [39], any serious event will be promptly reported to the ethics committee, and the affected participant will be withdrawn from the study.

### Data management

All original data will be comprehensively and legibly recorded and preserved in case report forms (CRFs), informed consent forms, and inspection reports. Researchers will be responsible for timely and accurate completion of the relevant information within the CRFs. A third-party data manager will oversee the CRFs throughout the study period. To guarantee data privacy and security, all study documents will be stored in a locked office. The Sichuan Provincial Acupuncture Clinical Medicine Research Center reserves the right to inspect study records and monitor the trial’s progress.

### Quality control

Prior to the trial commencement, all project staff will undergo specialized training on the study protocol and standard operating procedures (SOPs). Acupuncturists, who possess the necessary training, will be expected to adhere to a consistent approach regarding acupuncture prescription, needle angle, direction, manipulation, and stimulation. Additionally, to ensure researcher compliance, group meetings will be held twice a month for communication, study updates, and timely response to any challenges or unforeseen issues encountered during the trial.

### Statistical analysis

The data will be processed and analyzed by an independent statistician using IBM SPSS 26.0. An Intention-to-Treat (ITT) analysis approach will be adopted. Sensitivity analysis will use the Per-protocol (PP) analysis method. A two-sided *P-value* < 0.05 will indicate statistical significance. Descriptive statistics for continuous variables will include means and standard deviations (for normally distributed data) or medians and interquartile ranges (otherwise). Categorical variables will be presented as frequencies and percentages. Generalized estimating equations (GEE) will be used to assess the impact of the acupuncture intervention on outcome variables over time at baseline and weeks 12, 24, 48, and 60, with baseline measurement, time, group, and the group-by-time interaction included as covariates, as these factors may influence the efficacy of the acupuncture intervention. Patterns of missingness will be examined, and multiple imputation will be used assuming missing at random, with sensitivity analyses conducted where appropriate.

## Discussion

To explore the effective intervention measures of aMCI is one of the current research hotspots. Acupuncture has been widely used in non-drug therapies for various types of cognitive dysfunction, including MCI [40], postoperative cognitive impairment [41], post-stroke cognitive impairment [42], and vascular cognitive impairment [43], etc. The mechanism of acupuncture improving cognitive function may be related to enhancing hippocampal synaptic transmission, inhibiting neuroinflammation, regulating neurotransmitter levels, and alleviating oxidative stress [44–47]. Yang W et al.‘s bibliometric review suggested that acupuncture treatment of MCI can improve cognitive function [19]. A systematic review demonstrated that acupuncture appears effective for aMCI when used as an alternative or adjunctive treatment [48]. However, the study also suggested that more rigorously designed studies are needed to further verify this conclusion. According to our earlier preliminary clinical trial of acupuncture intervention in patients with aMCI, it was found that acupuncture has lasting but unclearly sustained positive effects. Since aMCI is a chronic disease that requires long-term observation, we will continue a long-term follow-up of 60 weeks in this trial to assess the sustained efficacy of acupuncture intervention.

Based on a network meta-analysis of diverse acupuncture interventions for MCI [22] manual acupuncture appears to be one of the most effective approaches. In this trial, we will employ manual acupuncture. The needles will be manipulated manually every 10 minutes to maintain the *deqi* sensation. Five acupoints will be selected for treatment: Taixi (KI 3), Dazhong (KI 4), Sanyinjiao (SP 6), Shenting (GV 24), and Baihui (GV 20). Studies suggest that KI 3 acupoint stimulation may enhance cerebral neuron activity in MCI patients [49,50]. Q Zhang et al. [51]. revealed that KI 4 is associated with influencing executive functions, emotional responses, and social behavior. SP 6 is a frequently used acupoint for various cognitive disorders [52]. Moreover, a data mining study has shown that SP6 is the core acupuncture point for acupuncture treatment of AD [53]. GV 24 and GV 20, located on the head and belonging to the governor meridian, play a vital role in influencing brain function, including cognition [22]. An animal experimental study found that electroacupuncture stimulation of GV 20 and GV 24 in mouse models can inhibit neuroinflammatory responses to prevent cognitive decline [54].

ADAS-Cog is a brief assessment instrument focused on multiple cognitive domains [55]. Previous studies support the use of ADAS-Cog in predicting the conversion to AD in aMCI population [56], and highlight its clinical utility in detecting those with aMCI [57,58]. Moreover, ADAS-Cog has been widely used as an outcome measure to assess therapeutic effect [59–61]. Over the years, anxiety and depression have been identified as risk factors for progression from aMCI to AD dementia [62]. It is now understood that depression, affecting approximately 52% of AD patients, can further exacerbate cognitive decline and increase mortality and suicide risk [63]. Furthermore, sleep plays a critical role in healthy cognition, including memory formation [64]. Sleep disorders may serve as both a potential risk factor for AD and worsen its pathological progression [65]. To comprehensively evaluate acupuncture’s effectiveness, we will explore its impact on cognitive function, emotional state, and sleep quality. The ADAS-Cog, HAMD, HAMA, and PSQI scales will be used to assess overall treatment outcomes. The control group will receive sham acupuncture. While debates persist about acupuncture’s effectiveness, with some attributing it to the placebo effect or arguing that sham acupuncture may not be entirely inert [66,67], this trial will implement rigorous blinding. Patients, researchers involved in recruitment, evaluation, and data analysis will remain unaware of group assignments, enhancing concealment and minimizing potential bias.

We acknowledge certain limitations within our trial. Firstly, acupuncture can cause mild pain and skin irritation. This discomfort may deter aMCI patients from consenting to treatment and completing the full course of the study. Secondly, due to the nature of the intervention, acupuncturists cannot be blinded to treatment allocation and potential effects. To minimize bias, acupuncturists will be instructed to avoid discussing treatment specifics with patients. Finally, due to the limitations of manpower and resources at present, only a standardized acupuncture will be studied, and further research is needed in the future.

## Conclusion

Overall, this randomized controlled trial will evaluate the long-term effect of acupuncture for treating patients with aMCI. This study aims to provide valuable insights and clinical guidance for the management of aMCI.

## Supporting information

S1 File SPIRIT checklist. (DOC)

S2 File Informed consent. (DOCX)

S3 File Clinical trial protocol. (DOCX)

S4 File Standard operating procedure for sealed envelopes for randomization. (DOCX)
