# Supplementary material for: Acupuncture for amnestic mild cognitive impairment: Study protocol for a multicenter, single-blinded, long-term, randomized controlled trial
Source: PLoS One. 2026 Apr 20;21(4):e0346717. doi: 10.1371/journal.pone.0346717 (PMC13094980; doi:10.1371/journal.pone.0346717)
Supplement: S2 File — (DOCX) [file pone.0346717.s002.docx]

**Informed consent**

**Dear participants**

Your doctor has diagnosed you with amnestic mild cognitive impairment. We will invite you to participate in a clinical trial entitled ''Study for the effect of acupuncture intervention on the patients with amnestic mild cognitive impairment based on the theory of "brain-kidney correlation''. Firstly, you need to understand that participation in this clinical trial is completely voluntary. Secondly, there is a big difference between the treatment received in clinical trials and the treatment received by your doctor outside the study. The research team needs to treat you in accordance with the requirements of the clinical trial protocol.

Before you decide whether to participate in this study, to help you understand the study and why the study, the procedures and duration of the study, the benefits, risks and discomfort that may bring to you after participating in the study. If you wish, you can also discuss it with your relatives and friends, or ask your doctor to give an explanation to help you make a decision. If you choose to participate in this clinical trial, you need to sign this informed consent. At the same time, you will receive a copy of the signed document for preservation.

This clinical trial was funded by the Sichuan Provincial Clinical Research Center for Acupuncture and Moxibustion. The Medical Ethics Committee of the Affiliated Hospital of Chengdu University of Traditional Chinese Medicine has conducted an ethical review and approval.

1. **Study background and purpose**

Mild cognitive impairment (MCI) is an intermediate stage between normal aging and dementia. It is characterized by objective evidence that cognitive function is deteriorating, but daily living ability is basically intact. MCI is considered to be a potential precursor of dementia. Studies have shown that with the rapid increase of the global aging population, the prevalence of MCI is even as high as 25.2%. And, the annual conversion rate of MCI to dementia is conservatively estimated to be 5% -17% per year, about 10 times that of the normal population. Therefore, the MCI stage is very important for the prevention of senile dementia. Preventive intervention in the MCI stage can effectively inhibit the occurrence and development of dementia. The most common clinical type of MCI is amnestic Mild Cognitive Impairment (aMCI), which is mainly manifested as memory impairment, with or without impairment of language, attention and visuospatial ability, etc. At present, the efficacy of drug therapy for aMCI patients is poor, so non-drug therapy is sought for control. Acupuncture therapy is a non-drug therapy commonly used in clinical treatment of aMCI. It has many advantages such as green, economic and non-toxic side effects. Nowadays, many systematic reviews / meta-analyses and randomized controlled trials have shown that acupuncture plays an important role in improving cognitive function, living ability and behavioral ability of aMCI patients. Some studies have shown that acupuncture can inhibit the aggregation of amyloid and tau protein and the apoptosis of nerve cells. Therefore, acupuncture therapy provides a method and idea from Chinese medicine for the treatment of aMCI.

The purpose of this study is to observe and compare the clinical efficacy and safety of acupuncture group and sham acupunctre groups for aMCI. The results of this study will be used to provide high-quality evidence-based evidence for the clinical application of acupuncture intervention in aMCI, so as to promote the improvement of clinical efficacy of acupuncture in the treatment of aMCI.

This study has been approved by the Medical Ethics Committee of the Affiliated Hospital of Chengdu University of Traditional Chinese Medicine. This study will be carried out simultaneously in 9 research centers in the province ( Hospital of Chengdu University of Traditional Chinese Medicine, the Fourth People's Hospital of Chengdu, the Sichuan Province People's Hospital, the Rehabilitation Hospital of Sichuan Province, the West China Hospital, Traditional Chinese Medicine Hospital of Pidu district, the Nanchong Second People's Hospital, Chengdu First People's Hospital and Traditional Chinese Medicine Hospital of Meishan) and 166 subjects are expected to participate voluntarily.

**2.Who will be invited to participate in this study ?**

(1) Patients who meet 8 following criteria will be included:

① diagnosis of aMCI according to the Jak/Bondi 2014 diagnostic criteria;

② fifty to eighty years of age, without limitation of sex;

③ course of disease≥3 months;

④ the Clinical Dementia Rating (CDR) score is 0.5;

⑤ the Hachinski Incheinic Score (HIS)≤4;

⑥education years≥8 (including vocational education) and correct understanding the completion of scales;

⑦ volunteering to cooperate and sign an informed consent form;

⑧ no contraindications to magnetic resonance imaging (MRI) scanning.

1. Patients will be excluded if they meet any of the following criteria:

① receiving treatment that interferes with cognitive function (e.g., the treatment of acute psychotic illness, such as memantine, rivastigmine, donepezil, oxiracetam, and sodium oligomannate capsules);

②a history of neurological conditions affecting cognitive function confirmed by examination, except in patients with suspected early AD, including Parkinson's disease, vascular dementia, brain tumour, traumatic brain injury, or other diseases which might lead to neurological injury and abnormal brain structure. Presence of a systemic diseases that could cause cognitive decline (such as anaemia, Hashimoto's encephalopathy, metabolic encephalopathy, hepatic encephalopathy, and renal encephalopathy);

③ the brain MRI showing infection or other local lesions, infarction in vital memory brain areas, multiple embolic infarctions, or severe white matter lesions (Fazekas score≥3);

④ a history of tumour, psychiatric illness (e.g., bipolar disorder, schizophrenia), or severe anxiety (Hamilton anxiety [HAMA] scale score≥29), major epression (Hamilton Depression [HAMD] scale score≥24);

⑤ severe haemorrhagic disease, bleeding tendency, or skin infection;

⑥ severe drug dependence, smoking, drug, or alcohol abus;

⑦ pregnant, lactating females, or potentially pregnant;

⑧ receipt of any acupuncture treatment or participation in other clinical trials within 6 months before enrollment.

**3.What will you need to do if you participate in the study ?**

This study is expected to last for 60 weeks. If you participate in this study, you first need to undergo a relevant examination to test whether you meet all the conditions for participating in the study. After screening, you may receive 12 weeks of treatment and 48 weeks of follow-up.

1. Before you are enrolled in the study, you will undergo the following checks to determine whether you can participate in the study :

The doctor will ask and record your medical history, clinical symptoms and signs, drugs being used and used in the past, and conduct physical examination and blood collection for blood test, brain MRI and other tests. All the above examinations are free of charge and will not adversely affect your health and condition.

1. If you meet the inclusion criteria through the above screening, the study will be conducted as follows:

①At the beginning of the study, the doctor will give you life adjustment advice based on your physical condition.

②On this basis, the doctor will decide which group of treatments you will receive according to a random plan. Patients who participated in this study were 1/2 likely to be assigned to either of these two different groups. Neither you nor your doctor can know and choose treatment in advance. The treatment methods of each group were safe and effective. During acupuncture, the needle will be retained for 30 minutes each time, 2 a week for 12 weeks, a total of 24 times.

The acupuncture needles used in this study were Huatuo brand disposable sterile acupuncture needles produced by Suzhou Medical Supplies Factory Co., Ltd., which had passed the inspection (standard number: GB2024-1994) and obtained the production license (production enterprise license: Jiangsu Food and Drug Administration Production License No.2001-0020, registration certificate No.2012 No.2270864).

③The observation period will last 60 weeks.

④During the treatment, you need to record your related symptoms in detail and truthfully. After the treatment, you should report the change of the condition to the doctor in time. The doctor will collect your medical history and make the necessary examination. You need to return to the doctor's clinic every 12 weeks during the study. Before treatment you will be physical examination, blood test, brain MRI, neuropsychological testing and so on. Doctors will also carry out other relevant laboratory examinations according to the specific clinical conditions. All the above examinations are free of charge, which will help doctors to understand your condition more comprehensively and carefully. These examinations are safe and will not have adverse effects on your health and condition.

(3) Other matters requiring your cooperation

You must visit the hospital according to the follow-up time agreed by the doctor and you. Your follow-up is very important because the doctor will determine whether the treatment you receive really works and give timely guidance.

You may not use drugs that treat aMCI during the study. If you need other treatment, please contact your doctor in advance.

1. **Potential benefits of participation in research**

Clinical studies have shown that acupuncture can effectively improve aMCI. The acupuncture treatment used in this study may have a better therapeutic effect on aMCI. Your condition may be improved or your symptoms may be alleviated. However, this still needs further verification and cannot be guaranteed to be effective for you. The acquisition of information in this study may help doctors and researchers to further confirm the therapeutic effect of acupuncture on aMCI for other patients with similar conditions.

1. **Possible risks and discomfort, inconvenience of participating in study**

In the process of acupuncture, you may have the feeling of acid, numbness, heaviness and swelling, which are the normal reactions of acupuncture. There may be adverse reactions after acupuncture, but less and mild, acupuncture may be due to your physical problems or emotional tension fainting phenomenon, stop acupuncture and proper rest can be alleviated. Hemorrhage, hematoma and other phenomena may occur after acupuncture, which can disappear after local compression. But if an infection occurs at the site of the needle, your doctor will treat it in time. If you experience any discomfort or new changes in your condition during the study period, or any unforeseen circumstances, whether related to acupuncture treatment or not, you should inform your doctor in a timely manner and he / she will make a judgment and give appropriate medical treatment. You need to be in the hospital on time during the study follow-up, do some checks, which may cause you trouble or inconvenience.

1. **About costs**

The costs of neuropsychological testing, blood examination (blood routine, biochemical items, homocysteine, folic acid, serum vitamin B12, five items of thyroid function, three items of antibody, five items of hepatitis B, glycosylated hemoglobin) and MRI examination during your participation in this study will be all free, and there will be free acupuncture treatment and 300 yuan of traffic compensation (issued according to the number of times).

Doctors will do their best to prevent and treat the possible harm caused by this study. If adverse events occur in clinical trials, the medical expert committee will identify whether they are related to acupuncture treatment or the trial process. In case of adverse reactions and injuries caused by acupuncture treatment, the aMCI research group of Chengdu University of Traditional Chinese Medicine) will pay the relevant treatment costs and corresponding economic compensation according to China's "Drug Clinical Trial Quality Management Specification."

If you merge other diseases required treatment and examination, will not be within the scope of free.

1. **Is personal information confidential?**

Your medical records (research medical records/case report form, laboratory sheets, etc.) will be completely stored in the hospital you visited. The doctor will record the results of the test on your medical record. Researchers and ethics committees will be allowed to access your medical records. Any public report about the results of this study will not disclose your personal identity. We will make every effort to protect the privacy of your personal medical data within the scope of the law.

1. **Voluntary choice to participate in study and drop out**

Whether to participate in the study depends entirely on your wishes. You can refuse to participate in this study, or withdraw from this study at any time during the study, which will not affect your medical treatment and rights.

For your best interests, your doctor or researcher may suspend your participation in the study at any time during the study. If you withdraw from the study for any reason, you may be asked about your acupuncture treatment. You may also be required to undergo a laboratory test and physical examination if doctors deems it necessary.

If an important event or information related to the subject occurs during the study, which may affect your willingness to continue to participate in the study, your doctor will notify you in time.

1. **How to get more information ?**

If you have any questions, suggestions or complaints about this study, please discuss with the study team in time. Contact information is available on the signature page. If you feel inconvenient to communicate with the research team, you can consult or complain to the Medical Ethics Committee of the Affiliated Hospital of Chengdu University of TCM, contact: 028-87783142.

**Informed Consent · Consent Signature Page**

**Clinical research project name:** Study for the effect of acupuncture intervention on the patients with amnestic mild cognitive impairment based on the theory of "brain-kidney correlation"

**Consent statement :**

1. I have read the above introduction to this study and have the opportunity to discuss and ask questions with doctors about this study.All the questions I have asked have been satisfactorily answered.
2. I know the possible risks and benefits arising from participating in this study.I know that participation in the study is voluntary, and I confirm that I have enough time to consider this, and I understand that:

(1) I can consult my doctor for more information.

(2) I can withdraw from this study at any time without discrimination or retaliation, and my medical treatment and interests will not be affected.

(3) I am also aware that if I quit the study, especially when I quit the study because of acupuncture, if I tell the doctor about the changes and complete the corresponding physical examination and physical and chemical examinations, it will be very beneficial to me and the whole study.

(4) If I need any other medication due to the condition change, I will ask my doctor in advance or tell the doctor truthfully after the event.

(5) I agree that Sichuan Acupuncture Clinical Medical Research Center, ethics committee or sponsor representative consult my research materials.

(6) I will obtain a signed and dated copy of the informed consent form.

Finally, I decided to agree to participate in this study and to ensure that I follow the doctor 's orders.

Patient signature： Date：

Telephone number：

I confirm that the details of the trial, including its rights and possible benefits and risks, were explained to the subject and given a signed copy of the signed informed consent form.

Investigator signature： Date：

Telephone number：

**患者知情同意书**

亲爱的患者：

医生已经初步诊断您患有遗忘型轻度认知障碍。我们现邀请您参加一项题为“基于“脑肾相关”理论的针刺干预遗忘型轻度认知障碍的临床疗效研究”的临床试验。首先，您需要了解的是参与这项临床试验是完全自愿的。其次，在临床试验中接受的治疗与在研究之外接受您的医生的治疗存在很大差异，研究团队需要遵照临床试验方案的要求对您进行治疗。

在您决定是否参加这项临床试验之前，请尽可能仔细阅读以下内容。它可以帮助您了解该项研究以及为何要进行这项研究，研究的程序和期限，参加研究后可能给您带来的益处、风险和不适等重要信息。如果您愿意，您也可以和您的亲属、朋友一起讨论，或者请医生给予解释，帮助您做出决定。如您选择参与这项临床试验，您需要签署这份知情同意书。同时，您将收到一份签署后文件的副本，以供保存。

该项目受四川省针灸临床医学研究中心资助，成都中医药大学附属医院医学伦理委员会已经对其进行伦理审查并批准。

1. **研究背景和研究目的**

轻度认知障碍（Mild cognitive impairment，MCI）是介于正常衰老和痴呆之间的一个中间阶段，其特征是有客观证据证实认知功能进行性恶化，但日常生活能力基本完好。MCI被认为是痴呆的潜在前驱症状，据研究表明随着全球老龄人口的急剧增加，MCI患病率甚至高达25.2%，同时，MCI向痴呆的年转化率保守估计为每年5%-17%，约为正常人群的10倍。因此，MCI期对于预防老年痴呆至关重要，故而在MCI阶段进行预防干预，才能有效抑制痴呆的发生发展。

MCI最常见的临床类型为遗忘型轻度认知障碍（amnestic Mild Cognitive Impairment，aMCI），主要表现为记忆力的损害，合并有或单独表现为语言、注意和视空间能力损害等。目前，针对aMCI患者药物治疗疗效欠佳，故而寻求非药物疗法进行控制。针灸疗法，是临床普遍运用治疗aMCI的一种非药物疗法，它具有绿色、经济、无毒副作用等诸多优势。目前许多系统评价/Meta分析和随机对照试验表明，针灸在改善aMCI患者的认知功能、生活能力、行为能力等方面扮演着重要角色。同时，有研究表明针灸可抑制淀粉样蛋白及tau蛋白的聚集及神经细胞的凋亡。故针灸疗法为治疗aMCI提供了一种来自于中国医学的方法和思路。

本研究的目的是为了观察比较针刺经穴、针刺非经非穴组对aMCI的临床疗效与安全性，其研究结果将用于为针刺干预aMCI的临床应用提供高质量的循证证据，以促进针灸临床治疗aMCI疗效的提升。

本试验已获得成都中医药大学附属医院医学伦理委员会批准，将在全省9个研究中心（成都中医药大学附属医院、成都市第四人民医院、四川省人民医院、四川省康复医院、四川大学华西医院、成都市郫都区中医医院、南充市第二人民医院、成都市第一人民医院、眉山市中医医院）同时进行，预计将有166名受试者参加。

1. **哪些人会被邀请参加这项研究？**

1. 同时符合以下条件的人，将被邀请参加该研究：

（1）符合aMCI诊断标准；

（2）年龄50~80岁，男女不限；

（3）病程超过3个月；

（4）临床痴呆量表（CDR）评分为0.5分；

（5）缺血指数量表（HIS）得分＜4分；

（6）受教育年限8年及以上（含职业教育及函授），能正确理解完成量表；

（7）自愿配合并签署知情同意书；

（8）体内无金属等核磁扫描禁忌物。

2. 如果您符合以下以下任意一点

（1）正接受干扰认知功能的治疗；

（2）经检查确认具有影响认知功能的神经系统疾病史（阿尔茨海默病早期疑似患者除外），包括帕金森病、血管性痴呆、脑肿瘤或脑外伤等导致神经损伤和其他大脑结构异常的疾病；具有导致认知能力下降的全身疾病等；

（3）经脑部MRI提示感染或其他局灶性损伤，多发性梗死，或位于脑重要记忆区域的梗死或严重的脑白质病变（Fazekas评分≥3分）；

（4）有肿瘤史、精神病史或严重的焦虑（HAMA≥29）和抑郁（HAMD≥24）者；

（5）有出血性疾病、出血倾向或严重皮肤感染者；

（6）严重药物依赖、吸烟、吸毒及酗酒者；

（7）孕妇、哺乳期妇女或疑似怀孕者；

（8）半年内接受过任何针刺治疗或参与过其他临床研究者。

您的研究医生会对您进行检查，以确认您是否可以参加本研究。

1. **如果参加研究将需要做什么？**

这项研究预期持续60周。如果您参加本次研究，您首先需要接受相关的检查以检验您是否符合参加这项研究的所有条件。筛选合格后，您将可能接受12周的治疗和48周的随访。

1. 在您入选研究前，您将接受以下检查以确定您是否可以参加研究：

医生将询问、记录您的病史、临床症状和体征、正在使用及过去使用过的药物，并进行认知测评、体格检查、血液检查、脑部MRI等检查。以上所有检查均免费，并且不会对您的健康和病情产生不良影响。

2. 若您通过以上筛查符合纳入标准，将按以下步骤进行研究：

（1）研究开始医生将根据您身体的具体情况提供生活调适建议。

（2）在此基础上，医生将根据随机方案决定您接受哪组治疗。参加这项研究的患者分别有1/2的可能性被分入这2个不同组中的任何一个。您和您的医生都无法事先知道和选择治疗方法。各组治疗方法均安全、有效。针刺时，每次留针30分钟，每周治疗2次，连续治疗12周，总共24次。

本研究中使用的针灸针为苏州医疗用品厂有限公司生产的华佗牌一次性使用无菌针灸针，已经过检查合格（标准号：GB2024-1994），获得生产许可（生产企业许可证：苏食药监生产许2001-0020号，注册证号：苏食药监械准字2012第2270864）。

（3）观察周期将持续60周。

（4）治疗期间您需要详细如实记录您的相关症状，治疗结束后及时向医生反映病情变化，医生将收集您的病史并做必要的检查。您需要在研究期间每12周到医生门诊复诊一次。治疗前将对您进行体格检查、血检、脑部MRI、认知测试等检查。医生还将根据临床具体情况，进行其他相关实验室检查，以上所有检查均免费，这些将有助于医生更加全面细致地了解您的病情，这些检查都很安全，不会对您的健康和病情产生不良影响。

3. 需要您配合的其他事项

您须要按医生和您约定的随访时间来医院就诊。您的随访非常重要，因为医生将判断您接受的治疗是否真正起作用，并及时进行指导。

在研究期间您不能使用治疗aMCI的药物。如您需要进行其它治疗，请事先与您的医生取得联系。

1. **参加研究可能的受益**

临床研究表明针刺可有效改善aMCI，本研究所采用的针刺治疗有可能对aMCI有较好治疗作用，您的病情有可能获得改善/您的症状有可能获得缓解；但这仍需要进一步的验证，不能保证对您肯定有效。本项研究信息的获取可能帮助医生和研究人员进一步确认针刺对aMCI的治疗作用，以用于有相似情况的其他患者。

1. **参加研究可能的风险和不适、不方便**

针刺过程中您可能会有酸、麻、重、胀的感觉，这均为针刺的正常反应。针刺后可能存在不良反应，但较少而轻微，针刺时可能因为您的体质问题或情绪紧张出现晕针现象，停止针刺和适当休息后可缓解；针刺后可能出现出血、血肿等现象，经局部按压后可消失；但如果针刺部位出现感染，您的医生会及时处理。如果在研究期间您出现任何不适，或病情发生新的变化，或任何意外情况，不管是否与针刺治疗有关，均应及时通知您的医生，他/她将对此作出判断并给与适当的医疗处理。您在研究期间需要按时到医院随访，做一些检查，这些都可能给您造成麻烦或带来不方便。

1. **有关费用**

您参加本项研究期间所做的与认知检查、血液检查（血常规、生化全项、同型半胱氨酸、叶酸、血清维生素B12、甲功五项、抗体三项、乙肝五项、糖化血红蛋白）及MRI的检查费用均为免费，并有免费针刺治疗和300元的交通补偿（按照次数进行发放）。

医生将尽全力预防和治疗由于本研究可能带来的伤害。如果在临床试验中出现不良事件，医学专家委员会将会鉴定其是否与针刺治疗或试验过程有关。如发生因针刺治疗引起的不良反应并造成伤害，成都中医药大学aMCI课题组将参照我国《药物临床试验质量管理规范》支付相关的治疗费用和相应的经济补偿。

如果您合并的其他疾病所需的治疗和检查，将不在免费范围之内。

1. **个人信息是保密的吗？**

您的医疗记录（研究病历/CRF、化验单等）将完整地保存在您所就诊的医院。医生会将化验检查结果记录在您的病历上。研究者、伦理委员会将被允许查阅您的医疗记录。任何有关本项研究结果的公开报告均不会披露您的个人身份。我们将在法律允许的范围内，尽一切努力保护您的个人医疗资料的隐私。

1. **可以自愿选择参加研究和中途退出研究**

是否参加研究完全取决于您的意愿。您可以拒绝参加此项研究，或在研究过程中的任何时间退出本研究，这都不会影响您的医疗待遇与权益。

出于对您的最大利益考虑，医生或研究者可能会在研究过程中随时中止您继续参加本项研究。如果您因为任何原因从研究中退出，您可能被询问有关您接受针刺治疗的情况。如果医生认为需要，您也可能被要求进行实验室检查和体格检查。

研究过程中如果发生与受试者相关的重要事件或信息，可能会影响您继续参加研究的意愿时，您的医生将及时通知您。

1. **怎样获得更多信息？**

如您对这项研究存在任何疑问、建议或投诉，请及时与研究团队讨论，联系方式见签字页。如您感觉不便与研究团队沟通，可向成都中医药大学附属医院医学伦理委员会进行咨询或投诉，联系电话：028-87783142。

知情同意书·同意签字页

**临床研究项目名称：**基于“脑肾相关”理论的针刺干预遗忘型轻度认知障碍的临床疗效研究

**同意声明：**

1、我已经阅读了上述有关本研究的介绍，而且有机会就此项研究与医生讨论并提出问题。我提出的所有问题都得到了满意的答复。

2、我知道参加本研究可能产生的风险和受益。我知晓参加研究是自愿的，我确认已有充足时间对此进行考虑，而且明白：

1. 我可以随时向医生咨询更多的信息。
2. 我可以随时退出本研究，而不会受到歧视或报复，医疗待遇与权益不会受到影响。
3. 我同样清楚，如果我中途退出研究，特别是由于针刺治疗的原因使我退出研究时，我若将我的病情变化告诉医生，完成相应的体格检查和理化检查，这将对整个研究十分有利。
4. 如果因病情变化我需要采取任何药物治疗，我会在事先征求医生的意见，或在事后如实告诉医生。
5. 我同意四川省针灸临床医学研究中心、伦理委员会或申办者代表查阅我的研究资料。
6. 我将获得一份经过签名并注明日期的知情同意书副本。

最后，我决定同意参加本项研究，并保证尽量遵从医嘱。

患者签名：_____________ _____年_____月_____日

手机号码：_____________

我确认已向患者解释了本试验的详细情况，包括其权利以及可能的受益和风险，并给其一份签署过的知情同意书副本。

医生签名：_____________ _____年 _____月 _____日

手机号码：_____________
