# Supplementary material for: Acupuncture for amnestic mild cognitive impairment: Study protocol for a multicenter, single-blinded, long-term, randomized controlled trial
Source: PLoS One. 2026 Apr 20;21(4):e0346717. doi: 10.1371/journal.pone.0346717 (PMC13094980; doi:10.1371/journal.pone.0346717)
Supplement: S3 File — (DOCX) [file pone.0346717.s003.docx]

**Study for the effect of acupuncture intervention on the patients with amnestic mild cognitive impairment based on the theory of "brain-kidney correlation"**

Research program

**Subject Undertaking Unit: the West China Hospital, the Fourth People's Hospital of Chengdu, the Sichuan Province People's Hospital, Traditional Chinese Medicine Hospital of Pidu district, the Nanchong Second People's Hospital, Traditional Chinese Medicine Hospital of Meishan, Chengdu First People's Hospital, the Rehabilitation Hospital of Sichuan Province and Hospital of Chengdu University of Traditional Chinese Medicine.**

1. **Research Objective**

This study takes patients with amnestic Mild Cognitive Impairment (aMCI) as the research object, and evaluates the effectiveness and safety of acupuncture intervention based on the theory of “brain-kidney correlation” in aMCI through a multi-center clinical randomized controlled study.

1. **Design types and principles**

**（1）Type of research design**

A prospective, multicenter randomized controlled, parallel design clinical study will be conducted.

**（2）Sample size and its calculation basis**

According to previous research and clinical experience, the average expected effect of the treatment group was 3.5, the control group was 1.5, and the standard deviation between the two groups was 3.5. The sample size was calculated using PASS software, n = 132 (α= 0.05,1-β= 0.90, bilateral). According to the 20 % expected dropout rate, the final test sample size of this study was 83 cases in each group, and a total of 166 aMCI subjects were included. The center plans to include 20 aMCI patients.

**（3）Random method**

Patients who met the inclusion criteria will be randomly assigned to 2 groups according to the ratio of 1:1. Random distribution will be performed by computer block randomization method. The generation and preservation of random numbers will be performed by a third party who do not participate in the experiment.

**（4）Blind design and implementation**

The whole experiment will be treated with blind method and blind evaluation. The patients will be separated and treated every time, and the grouping is not known. The curative effect will be also evaluated by a third party who do not know the grouping situation. In the data summary stage, blind statistical analysis will be used to separate researchers, clinical operators, efficacy evaluators and data statisticians.

**（5）Treatment group**

In this study, an acupuncture group will be established as a treatment group.

**（6）Control group**

In this study, a sham acupuncture group will be established as a control group.

1. **Research object**
2. **Cases source**

The study plan to recruit participants in 8 clinical sub-centers (the West China Hospital, the Fourth People's Hospital of Chengdu, the Sichuan Province People's Hospital, Traditional Chinese Medicine Hospital of Pidu district, the Nanchong Second People's Hospital, Traditional Chinese Medicine Hospital of Meishan, Chengdu First People's Hospital, the Rehabilitation Hospital of Sichuan Province and Hospital of Chengdu University of Traditional Chinese Medicine).

**（2）Diagnosis criteria**

The diagnostic criteria of MCI refer to the diagnostic criteria of Western medicine proposed by Jak/Bondi in 2014, using the delayed free recall (N5) and recognition (N7) scores of the auditory verbal learning test (AVLT), the total score of the animal fluency test (AFT), the total score of the Boston naming test (BNT), and the total time spent on the trail making test A and B, a total of 6 indicators: when 1 the scores of 2 tests in the same cognitive field are impaired (>1SD); or 2 Three cognitive domains (memory, execution, language) were diagnosed when one test score was impaired (>1SD), or 3 Daily living ability was impaired (FAQ>9). Among them, aMCI is the impairment of delayed free recall (N5) and/or recognition (N7) based on the diagnostic criteria of MCI.

**（3）Inclusion criteria**

Patients who meet 8 following criteria will be included:①a diagnosis of aMCI according to the Jak/Bondi 2014 criteria; ②age between 50 and 80 years, regardless of sex; ③ a disease course≥3 months; ④a Clinical Dementia Rating (CDR) score of 0.5; ⑤a Hachinski Ischemic Score (HIS)≤4;⑥≥8 years of education (including vocational) with the ability to understand and complete scales; ⑦willingness to participate and provide signed informed consent; ⑧no contraindications for magnetic resonance imaging (MRI) scanning.

**（4）Exclusion criteria**

Patients will be excluded if they meet any of the following criteria: ① receiving treatment that interferes with cognitive function (e.g., the treatment of acute psychotic illness, such as donepezil, memantine, oxiracetam, sodium oligomannate capsules, and rivastigmine); ②a history of neurological conditions affecting cognitive function confirmed by examination, except in patients with suspected early AD (including vascular dementia, Parkinson's disease, traumatic brain injury, brain tumor or other diseases which might lead to neurological injury and abnormal brain structure). Presence of systemic diseases that could cause cognitive decline (such as hepatic encephalopathy, Hashimoto's encephalopathy, metabolic encephalopathy, renal encephalopathy, and anemia); ③ the brain MRI showing infection or other local lesions, infarction in vital memory brain areas, multiple embolic infarctions, or severe white matter lesions (Fazekas score≥3); ④ a history of psychiatric illness (e.g., bipolar disorder, schizophrenia), severe anxiety (Hamilton anxiety [HAMA] scale score≥29), major depression (Hamilton Depression [HAMD] scale score≥24), or tumor; ⑤severe skin infection, hemorrhagic disease, or bleeding tendency; ⑥severe drug dependence, drug, smoking, or alcohol abuse; ⑦ pregnant, potentially pregnant, or lactating females; and ⑧receipt of any acupuncture treatment or participation in other clinical trials within 6 months before enrollment.

**（6）Withdrawal criteria and handling**

①Withdrawal criteria

Patients who do not meet the inclusion criteria and are mistakenly enrolled; patients have poor compliance and withdrew from the treatment course on their own; patients who utilize prohibited treatments or independently modify their treatment plan as outlined in the protocol; patients who experience significant life events or circumstances that make continued participation unsuitable for their well-being.

②Handling

After the subject is removed, the attending physician should contact the subject as much as possible through home visits, appointment phone calls, letters, etc., to inquire about the reasons, record the last treatment time, and complete all the assessment items that can be completed; For cases that withdraw from the trial due to adverse reactions or ineffective treatment, the attending physician should take corresponding measures based on the actual situation of the subjects; Fill in the "Summary of Treatment Completion" and "Clinical Trial Completion Status" of CRF; Once they are enrolled in the group in sequence, they become the subjects of the trial observation, regardless of the subsequent diagnosis and whether the treatment is complete; All excluded and dropped cases were analyzed for intention-to-treat analysis after the end of the trial.

**（7）Discontinuation of study cases**

Serious adverse reactions in the study, should not continue to participate in the study; During the study period, the subjects have serious complications or deterioration of the disease, and emergency measures should be taken; During the study period, the patient's condition changed, and the attending physician of neurology suggested that the clinical researcher could not continue; The subjects propose to withdraw from the clinical study halfway; patients do not cooperate, do not obey the treatment, the clinician repeatedly explained ineffective; Researchers should record the reasons and time of withdrawal from the study in detail, and those who have more than 1/2 courses of treatment should enter the efficacy statistics.

1. **Treatment plan and control measures**

**（1）Grouping and acupoint selection**

① Acupuncture group

The following acupoints will be used: bilateral Taixi (KI 3), bilateral Dazhong(KI 4), bilateral Sanyinjiao (SP 6), Shenting (GV 24), and Baihui (GV 20) (Table 3). Taixi (KI 3), posterior to the medial malleolus, in the depression between tip of the medial malleolus and tendo calcaneus. Dazhong (KI 4), under the medial malleolus of the foot, the medial anterior depression of the achilles tendon attachment. Sanyinjiao (SP 6), on the inside of the calf, 3 cun above the tip of the medial malleolus, posterior border of the medial border of the tibia. Shenting (GV 24), 0.5 cun directly above the midpoint of the anterior hairline. Baihui (GV 20), 5 cun directly above the midpoint of the anterior hairline, at the midpoint of the line connecting the apexes of the two auricles. After skin disinfection, acupuncturists will insert single-use sterile needles (Hwato, Suzhou, China; 0.25×25 mm) into the acupoints. A uniform reinforcing-reducing method will be employed, involving twisting, thrusting, and rotation to elicit the deqi sensation within the patient's tolerance. Needles will be manually manipulated every 10 minutes to maintain the deqi sensation, with a twisting angle of 90-180 degrees, a frequency of 60-90 times/minute, and a lifting and inserting amplitude of 3-5 mm.

②Sham acupuncture group

The non-acupoint selection protocol was developed in our previous clinical trial. Skin disinfection will precede needle insertion. Non-acupoint 1, at the medial arm on the anterior border of the insertion of the deltoid muscle at the junction of deltoid and biceps muscles (On the right arm). Non-acupoint 2, at the medial arm on the anterior border of the insertion of the deltoid muscle at the junction of deltoid and biceps muscles (On the left arm). Non-acupoint 3, half way between the tip of the elbow and axillae (On the right arm). Non-acupoint 4, Half way between the tip of the elbow and axillae (On the left arm). Non-acupoint 5 , ulnar side, half way between the epicodylus medialis of the humerus and ulnar side of the wrist (On the right arm). Non-acupoint 6, ulnar side, half way between the epicodylus medialis of the humerus and ulnar side of the wrist (On the left arm). Non-acupoint 7, edge of the tibia 1-2 cm lateral to the Zusanli (ST36) horizontally (On the right leg). Non-acupoint 8, edge of the tibia 1-2 cm lateral to the Zusanli (ST36) horizontally (On the left leg). It will involve shallow acupuncture 0.3-0.5 cun, inserted perpendicularly) at eight non-acupoints.Skin disinfection will precede needle insertion. However, there will be no stimulation, manipulation, or attempts to elicit the deqi sensation.

**（2）Materials**

Acupuncture needle selection of suzhou medical supplies factory, the production of Hwato brand disposable acupuncture needle, production enterprise license: su food and drug administration production license 2001-0020, registration number: su food and drug administration (quasi) word 2012 no.2270864. Specifications are 0.25×13mm, 0.25×25mm.

**（3）Acupuncture operation**

① Disinfection: The doctor's hand disinfection: Before acupuncture, the doctor first rinse the hand with soapy water, and then wipe it with 75 % alcohol cotton ball before holding the needle. Acupuncture site disinfection: acupuncture site with 75 % alcohol cotton ball wipe disinfection, wipe should be from the center to the outside of the circle disinfection.

②Needle insertion: During acupuncture, both hands are used for acupuncture, and the claw cutting method is selected according to the characteristics of the location of the acupoint. The direction and depth of acupuncture are strictly in accordance with the requirements of acupoint acupuncture operation.

③Retaining the needle: The two groups are needled for 30 minutes after acupuncture.

④withdrawal of needles: The left thumb and the forefinger hold the disinfected dry cotton ball and gently pressed the acupuncture site. The right hand hold the needle for a slight slight twist, and the needle is slowly lifted to the subcutaneous tissue, static for a moment, and then the needle was taken out. After the needle is taken out, the needle hole is pressed to prevent bleeding.

**（4）Course of treatment**

The frequency of acupuncture treatment is 2 times/week, the treatment period is 12 weeks, the follow-up period was 48 weeks, and the overall research period is 60 weeks.

**（5）Provisions for combined medication**

During clinical trials, patients will be allowed to perform basic treatments such as blood pressure control, blood glucose control, and other supportive treatments, and the time, dose, frequency, and post-medication reactions were recorded. At the same time, it is suggested that the subjects should try not to use other drugs that affect cognitive function, acupuncture, massage and other methods. If the symptoms are serious, the clinicians should formulate the corresponding treatment plan and record the time, dose, frequency and reaction after medication.

1. **Observation indicators**
2. **General project:**

Clinical trial institution, subject number, test start date, address, contact phone, etc.

1. **Biological indicators**

①demographic characteristics: gender, age, height, weight, occupation, marital status, education; ②Vital signs: body temperature, heart rate, respiration, blood pressure; ③ Basic medical history collection, routine physical examination, traditional Chinese medicine syndrome type; ④ Laboratory examination: blood routine, biochemical items, homocysteine, folic acid, serum vitamin B12, five items of thyroid function, three items of antibody ( hepatitis C, syphilis, AIDS ), five items of hepatitis B, glycosylated hemoglobin. The above indicators were recorded at the time of enrollment.

1. **Diagnostic indicators**

The chief complaint, clinical manifestations, medical history, neuropsychological scale examination and other examinations were performed, and the disease will be diagnosed by the attending doctor.

**（4）Outcomes**

①Primary outcome measures

Cognitive function will be measured using the ADAS-Cog scale. The primary outcomes will be the changes in ADAS-Cog score at baseline and after treatment (the 12th weeks) and at baseline and 60th weeks.

②Secondary outcome measures

The secondary outcome measures include the following: The overall cognitive function will be measured using the Alzheimer's Disease Assessment Scale-Cognitive (ADAS-cog) scale, assessed at the12th, 24th, 36th, 48th, and 60th weeks; The memory function will be measured using the word recall and word recognition improvement tasks of ADAS-Cog scales at the12th, 24th, 36th, 48th, and 60th weeks; The emotional disorders will be measured using the HAMA Scale and HAMD Scale at the12th, 24th, 36th, 48th, and 60th weeks; and The sleep quality will be measured using the Pittsburgh Sleep Quality Index (PSQI) at the12th, 24th, 36th, 48th, and 60th weeks.

**（5）Research Evaluation Indicators**

Combined medication, compliance, shedding and elimination will be recorded during and at the end of the trial.

**（6）Safety observation**

Adverse events caused by acupuncture will be recorded at any time.

1. **Observation and analysis of adverse events**

The adverse events that occurred during the study must be recorded and reported, and the subjects should be treated in a timely and reasonable manner. The researcher should explain to the subjects that the subjects ( or family members ) are required to truthfully respond to the changes in the condition after treatment. Doctors avoid guided questions. In the observation of curative effect, pay attention to the observation of adverse events. When adverse events occur, the subjects are recorded and treated in a timely and appropriate manner until they are completely restored to normal.

Regardless of whether the adverse events are related to the treatment method of this study, they should be recorded in detail, including the time of occurrence of adverse events, symptoms, signs, degree, duration, laboratory examination indicators, treatment methods and results, process, follow-up time, etc., and analyze the causes of adverse events.

1. **Efficacy and safety evaluation**

**（1）Overall efficacy evaluation**

According to the situation of the subjects after treatment, the improvement of ADAS-cog score before and after treatment will be evaluated. After treatment, the subjects will evaluate the effect of acupuncture treatment.

**（2）Security assessment**

① Incidence of adverse events

Adverse events (name, symptoms), date of occurrence, severity, treatment measures, outcome, etc.during the treatment of different treatment regimens will be recorded, and their correlation with treatment will be evaluated.

②Safety evaluation

The adverse events of different treatment regimens in this study will be statistically compared to make a safety evaluation of different treatment regimens.

The occurrence time, symptoms, severity, treatment measures and disappearance time of adverse events will be judged in detail. When adverse events are found, clinicians will judge whether to suspend the test according to the condition. In severe cases, they will be reported to the College of Acupuncture and Moxibustion and Ethics Committee of Chengdu University of Traditional Chinese Medicine within 24 hours, and relevant records will be made at the same time.

Safety evaluation according to the following classification :

Level 1: safe, without any adverse reactions;

level 2: relatively safe, with mild adverse reactions, without any treatment can continue treatment;

level 3: There are safety problems, moderate adverse reactions, and treatment can be continued after treatment;

level 4: This study is discontinued due to adverse reactions.

**基于“脑肾相关”理论的**

**针刺干预遗忘型轻度认知障碍的**

**临床疗效研究**

**研** **究** **方** **案**

**课题承担单位: 成都中医药大学、成都中医药大学附属医院、成都市第四人民医院、四川省人民医院、四川省康复医院、四川大学华西医院、成都市郫都区中医医院、南充市第二人民医院、眉山市中医医院**

**一、研究目的**

本研究以遗忘型轻度认知障碍（amnestic Mild Cognitive Impairment ，aMCI）患者为研究对象，通过多中心的临床随机对照研究评价基于“脑肾相关”理论的针刺干预aMCI的有效性及安全性。

**二、设计类型与原则**

**1. 研究设计类型**

采用前瞻性、多中心随机对照、平行设计的临床研究。

**2. 样本量及其计算依据**

根据之前的研究和临床经验，治疗组的预期效果平均值为 3.5 ，对照组为 1.5 ，两组之间的标准差为 3.5。使用 PASS 软件计算样本量，n = 132(α = 0.05，1 — β = 0.90，双侧）。按 20%预计脱失率，本研究最终试验样本量为每组 83 例，总共纳入 166例 aMCI 受试者。本中心计划纳入 20 名aMCI 患者。

**3. 随机方法**

对符合纳入标准的病人，按照 1 ：1 的比例随机分配到本试验的 2 个组。随机分配采用计算机区组随机方法，随机号码的产生、保存均由不参与试验的第三方进行。

**4. 盲法设计及实施**

整个试验采用盲法治疗和盲法评价，患者每次治疗进行分离治疗，并不知分组情况；测评也由不知分组情况的第三者进行疗效评价；资料总结阶段采用盲法统计分析，实行研究者、临床操作者、疗效评价者和数据统计者的分离。

**5. 治疗组**

本研究设立了针刺经穴组作为治疗组。

**6. 对照组**

本研究设立了针刺非经非穴组作为对照组。

**三、研究人群**

**1. 病例来源**

该研究计划在 8 个临床分中心（成都中医药大学附属医院、成都市第四人民医院、四川省人民医院、四川省康复医院、四川大学华西医院、成都市郫都区中医医院、南充市第二人民医院、眉山市中医医院）及四川省各社区和福利院进行受试者的招募。

**2. 诊断标准**

MCI 诊断标准参照Jak/Bondi2014 年提出的西医诊断标准，采用听觉词语学习测验（AVLT）的延迟自由回忆（N5）与再认（N7）得分、动物流畅性测验总分（AFT）、 Boston 命名测验总分（BNT）、连线测验 A 与 B 的总耗时数，共 6 项指标：当①同一认知领域的 2 个测验得分受损（>1SD）；或②3个认知领域（记忆、执行、语言）均有 1 个测验得分受损（>1SD）时，或③日常生活能力受损（FAQ>9）时，即可诊断。其中aMCI 为在 MCI 的诊断标准基础上，有延迟自由回忆（N5）和/或再认（N7）的受损。

**3. 纳入标准**

aMCI 相关纳入标准如下：

（1）符合 aMCI 诊断标准；

（2）年龄 50~80 岁，男女不限；

（3）病程超过 3 个月；

（4）临床痴呆量表（CDR）评分为 0.5 分；

（5）缺血指数量表（HIS）得分＜4 分；

（6）受教育年限 8 年及以上（含职业教育及函授），能正确理解完成量表；

（7） 自愿配合并签署知情同意书；

（8）体内无金属等核磁扫描禁忌物。

注：同时符合以上标准的患者，方可纳入本研究。

**4. 排除标准**

（1）正接受干扰认知功能的治疗（如急性精神疾病发作的治疗，如美金刚、利伐斯的明、多奈哌齐、奥拉西坦、甘露特纳等的治疗）；

（2）经检查确认具有影响认知功能的神经系统疾病史（阿尔茨海默病早期疑似患者除外），包括帕金森病、血管性痴呆、脑肿瘤或脑外伤等导致神经损伤和其他大脑结构异常的疾病；具有导致认知能力下降的全身疾病，如贫血、桥本脑病、代谢性脑病、肝性脑病、肾性脑病等；

（3）经脑部 MRI 提示感染或其他局灶性损伤，多发性梗死，或位于脑重要记忆区域的梗死或严重的脑白质病变（Fazekas 评分≥3 分）；

（4）有肿瘤史、精神病史（如双相情感障碍、精神分裂症）或严重的焦虑（HAMA≥29）和抑郁（HAMD≥24）者；

（5）有出血性疾病、出血倾向或严重皮肤感染者；

（6）严重药物依赖、吸烟、吸毒及酗酒者；

（7）孕妇、哺乳期妇女或疑似怀孕者；

（8）半年内接受过任何针刺治疗或参与过其他临床研究者。

注：凡符合上述任何一条的患者，即予以排除。

**5. 研究病例的剔除、脱落与处理**

**5.1 剔除与脱落标准：**

（1）凡不符合纳入标准而被误入的病例应予剔除；

（2）受试者依从性差，疗程中自行退出者；

（3）合并使用本方案禁止使用的治疗方法，或自行中途更换治疗方法；

（4）出现严重疾病或家庭发生重大变故，不宜继续持续接受治疗的病例。

**5.2 剔除与脱落病例的处理：**

（1）当受试者脱落后，主管医生应采取登门、预约电话、信件等方式尽可能与受试者联系询问理由，记录最后一次治疗时间，完成所能完成的评估项目；

（2）因不良反应、治疗无效而退出试验病例，主管医生应根据受试者实际情况采取相应措施；

（3）填写 CRF 的“治疗完成情况总结” 、“临床试验完成情况”；

（4）一旦按序入组后，即成为试验观察的对象，不管以后诊断如何及治疗是否完整；

（5）所有剔除、脱落病例于试验结束后进行意向性分析。

**6. 研究病例的中止**

（1）研究中出现严重不良反应，不宜继续参加研究者；

（2）研究期间受试者出现严重并发症或病情恶化，需采取紧急措施者；

（3）研究期间患者病情变化，神经内科主诊医生建议不能继续本临床研究者；

（4）受试者中途提出退出临床研究；

（5）患者不合作、不服从治疗，经临床医生反复解释无效；

（6）研究者应详细记录退出研究的原因及时间，已超过 1/2 疗程者应进入疗效统计。

**四、治疗方案和对照措施**

**1. 分组及选穴**

（1）治疗组：

本研究选穴、定位及操作情况如下：

太溪（KI 3）：在足踝区，内踝尖与跟腱之间凹陷中；

大钟（KI 4）：在足内侧内踝后下方，当跟腱附着部的内侧前方凹陷处；

三阴交（SP 6）：在小腿内侧，内踝尖上 3 寸，胫骨内侧缘后际；

神庭（GV 24）：在头部，前发际正中直上 0.5 寸；

百会（GV 20）：在头部，前发际正中直上 5 寸。

操作：皮肤常规消毒后，针刺入穴位，之后采用捻转、提插手法，捻转的角度在 90～180 度之间，频率在 60～90 次/分钟之间；提插的幅度在 0.3～0.5 厘米之间，频率在60～90 次/分钟之间，捻转、提插幅度和频率采用均等的手法使得气，以患者耐受为度，每 10 分钟行针一次以保持得气感。

（2）对照组：

根据本课题组前期研究及相关文献记载的选取 8 个非经非穴点：

非经非穴点 1 、2 ：位于左/右上臂内前缘，肱二头肌与三角肌的结合处；

非经非穴点 3 、4 ：位于左/右上臂，腋窝与肘尖连线的中点；

非经非穴点 5、6：位于左/右前臂，腕掌侧横纹尺侧端与肱骨内上髁连线的中点；

非经非穴点 7 、8 ：位于左/右小腿部胫骨前缘处，即足三里穴（ST 36）横向旁开 1 至 2cm。

操作：皮肤常规消毒后，进针则垂直于皮肤刺入 0.3~0.5 寸，进针后不进行行针手法，不追求得气感。

**2. 试验材料**

毫针：针灸针选用苏州医疗用品厂有限公司生产的华佗牌一次性针灸针，生产企业许可证：苏食药监械生产许 2001-0020号，注册证号：苏食药监械（准）字2012第2270864号。规格是0.25×13mm 、0.25×25mm。

**3. 针刺基本操作**

（1）消毒

医者手消毒：针刺前，医者先用肥皂水将手洗刷干净，再用75%的酒精棉球擦拭后，方可持针操作。

针刺部位消毒：针刺部位用75%的酒精棉球擦拭消毒，擦拭时应从中心向外绕圈消毒。

（2）进针与行针

针刺时，均采用双手进针法，根据穴位所在部位的特点选择爪切法，针刺的方向、深度严格按照穴位针刺操作的要求。

（3）留针

两组在针刺后留针30分钟。

（4）出针

左手拇、食两指持消毒干棉球轻轻按于针刺部位，右手持针作轻微的小幅度捻转，并顺势将针缓缓提至皮下，静留片刻，然后出针，出针后按压针孔以防出血。

**4. 疗程**

针刺治疗频率为 2 次/周，治疗周期为 12 周，随访期为 48 周，整体的研究周期为 60 周。

**5. 合并用药的规定**

在临床试验期间，允许患者进行如血压控制、血糖控制和其他支持性治疗等基础治疗，记录用药的时间，剂量，次数，用药后反应等。同时建议受试者尽量不使用其他影响认知功能药物治疗及针灸、按摩、敷贴、熏蒸等方法，如果症状严重，由临床医生制定相应的治疗方案并记录用药的时间，剂量，次数，用药后反应等。

**五、观察指标**

**1. 一般项目**

临床试验机构，受试者编号，试验开始日期，住址，联系电话等。

**2. 生物学指标**

（1）人口学体征：性别、年龄、身高、体重、职业、婚姻状况、受教育情况。

（2）生命体征：体温，心率，呼吸，血压。

（3）基本病史采集，常规查体，中医证型。

（4）实验室检查：血常规、生化全项、同型半胱氨酸、叶酸、血清维生素 B12、甲功五项、抗体三项（丙肝、梅毒、艾滋）、乙肝五项、糖化血红蛋白。以上指标于入组时记录。

**3. 诊断学指标**

主诉及临床表现、病史、神经心理量表检查等检查，并由主诊医生进行疾病诊断。

**4. 疗效指标**

（1）主要指标：

阿尔茨海默病评价量表-认知分量表（Alzheimer's Disease Assessment Scale - Cognitive ，ADAS-cog）：入组第 12 周较基线期的认知功能变化情况。

（2）次要指标

①认知功能相关指标：

入组第 12 周及随访期第 24、36、48、60 周时 aMCI 患者 ADAS-cog：整体认知功能改善情况。

②记忆功能相关指标：

入组第 12 周及随访期第 24 、36 、48 、60 周时 aMCI 患者 ADAS-cog 单词回忆及单词辨认（再认）改善情况。

③情绪障碍：

汉密尔顿焦虑量表（Hamilton Anxiety Scale ，HAMA） 、汉密尔顿抑郁量表（Hamilton Depression Scale ，HAMD）：入组第 12 周及随访期第 24 、36 、48 、60周时 aMCI 患者情绪改善状态情况。

④睡眠情况：

匹茨堡睡眠质量指数量表（Pittsburgh Sleep Quality Index ，PSQI）：入组第 12周及随访期第 24 、36 、48 、60 周时 aMCI 患者的睡眠改善情况。

⑤有效率：入组第 12 周及随访期第 24 、36 、48 、60 周时 aMCI 患者的治疗有效率改善情况。

⑥其他指标：

药物使用情况、健康教育完成度评价、针灸期待值评价、治疗满意度评价。

注：每项评分由各中心不知治疗方案的受试者主管医师负责评价。为排除干扰因素，每位受试者均由同一名医师评价。

**5. 研究评价指标**

合并用药、依从性、脱落与剔除等，于试验中及结束时记录。

**6. 安全性观察**

针刺引起的不良事件随时记录。

**六、不良事件观察与分析**

对研究过程中出现的不良事件必须进行记录和报告，并对受试者进行及时合理的处理。研究者要向受试者说明，要求受试者（或家属）如实反应治疗后的病情变化。医生避免向导性提问。在观察疗效时，注意观察不良事件。当出现不良事件时，受试者会得到记录和及时妥善的处理，直至完全恢复正常。

无论不良事件是否与本研究治疗方法有关，均应详细记录，包括不良事件出现时间、症状、体征、程度、持续时间、实验室检查指标、处理方法与结果、经过、随访时间等，并且分析出现不良事件的原因。

**七、疗效与安全性评价**

**1. 受试者的总体疗效评价**

（1）根据受试者治疗后情况，对治疗前后 ADAS-cog 评分的改善情况作出评价；

（2）经治疗后，受试者对经针刺治疗的效果作出评价。

**2. 安全性评价**

（1）不良事件出现率

记录不同治疗方案治疗过程中出现的不良事件（名称、症状），出现日期、严重程度、处理措施、转归等，评价其与治疗的相关性。

（2）安全性评价

就本次研究中不同治疗方案出现的不良事件，进行统计比较，做出不同治疗方案的安全性评价。

详细判断不良事件的出现时间、症状、严重程度、处理措施及消失时间，发现不良事件时，临床医师依据病情判断是否中止试验，严重者 24 小时内报成都中医药大学针灸推拿学院和伦理委员会，并同时做好相关记录。

安全性评价按照以下分级：

1 级：安全，无任何不良反应；

2 级：比较安全，有轻度不良反应，不需任何处理可继续治疗；

3 级：有安全性问题，有中等程度不良反应，做处理后可继续治疗；

4 级：因不良反应中止本研究。
