# Supplementary material for: Acupuncture for amnestic mild cognitive impairment: Study protocol for a multicenter, single-blinded, long-term, randomized controlled trial
Source: PLoS One. 2026 Apr 20;21(4):e0346717. doi: 10.1371/journal.pone.0346717 (PMC13094980; doi:10.1371/journal.pone.0346717)
Supplement: S4 File — (DOCX) [file pone.0346717.s004.docx]

**Standard Operating Procedure for Sealed Envelopes for Randomization**

1. Preparation and Generation of Envelopes​

An independent statistician (not involved in participant recruitment, assessment, or intervention) generated the randomization sequence using SAS® version 9.4 (SAS Institute Inc., Cary, NC, USA). The sequence was finalized before patient enrollment. The sequence and corresponding group assignments (AG or SA) were sealed in a series of sequentially numbered, opaque envelopes with tamper-evident seals. Each envelope was labeled externally only with a unique serial number. A cardboard insert was placed inside each envelope to prevent show-through, ensuring the content could not be viewed from the outside.

2. Storage and Distribution of Envelopes​

The complete set of envelopes was kept by an independent statistician, stored in a locked filing cabinet. Based on the projected number of enrollments per center, envelopes corresponding to specific serial number ranges were distributed to the nine study centers. Upon receipt, each center verified the sequential order of the serial numbers and signed a transfer record. At each center, the envelopes were stored securely and managed by a designated envelope custodian who was not involved in patient recruitment or eligibility assessment.

3. Procedure for Envelope Use and Opening​

Prior to randomization, all participants were informed of the possible treatment groups to which they could be assigned. After the investigator confirmed that a patient met all inclusion/exclusion criteria and provided signed informed consent, the envelope custodian was notified. The custodian retrieved the corresponding sequentially numbered envelope (e.g., envelope No. 001 for the first enrolled patient) and opened it via video conference in the presence of the investigator. Once opened, the group assignment code inside was recorded in the case report form, concluding allocation concealment. The opened envelope and its contents were archived for future monitoring or audit verification. It was strictly prohibited to open any envelope in advance or without authorization.
